# Scorpion and Frog Organ Lysates are Potential Source of Antitumour Activity

**DOI:** 10.31557/APJCP.2020.21.10.3011

**Published:** 2020-10

**Authors:** Morhanavallee Soopramanien, Naveed Ahmed Khan, Sumayah Abdelnasir Osman Abdalla, K Sagathevan, Ruqaiyyah Siddiqui

**Affiliations:** 1 *Department of Biological Sciences, Sunway University, Bandar Sunway, Malaysia. *; 2 *Department of Biology, Chemistry and Environmental Sciences, College of Arts and Sciences, American University of Sharjah, University City, Sharjah, United Arab Emirates. *

**Keywords:** Scorpion, anticancer effects, frog, cytotoxicity, growth inhibition

## Abstract

**Objectives:**

It is noteworthy that several animal species are known to withstand high levels of radiation, and are exposed to heavy metals but rarely been reported to develop cancer. For example, the scorpion has been used as folk medicine in ancient civilizations of Iran and China, while amphibian skin is known to possess medicinal properties. Here, we elucidated the anti-tumour activity of the scorpion (Uropygi) and frog (Lithobates catesbeianus).

**Materials and Methods:**

Animals were procured and their organ lysates and sera were prepared and tested against Michigan Cancer Foundation-7 breast cancer (MCF-7), prostate cancer (PC3), Henrietta Lacks cervical cancer (HeLa), and normal human keratinocyte cells. Exoskeleton, appendages and hepatopancreas were dissected from the scorpion, whereas liver, lungs, heart, oviduct, gastrointestinal tract, gall bladder, kidneys, eggs and sera were collected from frog and organ lysates/sera were prepared. Growth inhibition assays and cytotoxicity assays were performed.

**Results:**

Appendages, exoskeleton lysates, and hepatopancreas from scorpion exhibited potent growth inhibition, and cytotoxic effects. Furthermore, lungs, liver, gastrointestinal tract, heart, oviduct, kidneys, eggs, and sera from frog displayed growth inhibition and cytotoxic effects.

**Conclusion:**

Organ lysates, sera of scorpion, and amphibians possess anti-tumour activities. This is a worthy area of research as the molecular identity of the active molecule(s) together with their mechanism of action will lead to the rational development of novel anticancer agent(s).

## Introduction

Cancer is a condition that remains a major threat to humans, being the 2nd leading cause of death worldwide. Cancer is described as a condition whereby cells undergo uncontrolled growth, defeating the natural cellar mechanisms of cell division (Jeyamogan et al., 2019; Mitra et al., 2015; Soopramanien et al., 2019; Sudhakar, 2009). Despite various treatment options available, such as radiotherapy, surgery, and chemotherapy, cancer remains a threat with the increasing number of incidence and deaths. For example, in 2018 alone, 18.1 and 9.6 million cases accounted for reported cancer incidence and deaths respectively (Bray et al., 2018). The morbidity and mortality have remained high despite our advances in chemotherapeutic treatments and supportive care. Furthermore, the increasing resistance of cancer cells to currently available anticancer agents and side effects due to these treatment options are additional concerns. Hence there is a clear need to ameliorate the current available treatments or to introduce new treatment options or anticancer agents to target this condition (Zugazagoitia et al., 2016). In particular, the problem of drug resistance continues to be a key factor in the recurrence of cancer in patients (Vasan et al., 2019). For instance, regardless of various drug combinations and regimens, patients with advanced breast cancer, similarly to other solid tumours, inescapably acquire resistance to the treatment (Moiseenko et al., 2017). Moreover, in many occurrences, tumours such as renal cancer, malignant melanoma and hepatocellular carcinoma often display inherent resistance to therapy, even without previous exposure to the anticancer agents (Nikolaou et al., 2018). Moreover, as cancer is a multifaceted and complex systemic disease, the need for novel chemotherapeutic agents that can block or target several steps of cancer cell characteristics, or modulate immune cells, and are less toxic to healthy tissues are required. Accordingly, there is a necessity to source anticancer agents from novel sources to discover potential anti-tumour molecules. 

Previous research has focused on plants as a source of anticancer agents. However, it is noteworthy that several animal species are well known to withstand high levels of radiation, reside in unsanitary environments, feed on rotten meat or germ infested environments, and are exposed to heavy metals but rarely been reported to develop cancer. They have been inhabiting the planet over millions of years and commonly used in folk medicine. These species must have developed mechanisms to defend themselves against cancer development. For example, the scorpion has been used as folk medicine in ancient civilizations of Iran and China, while amphibian skin is known to possess medicinal properties. These findings suggest that animals are also a useful source of novel agents. To this end, previous studies have shown potent anticancer activity from bioactive molecules isolated from animals such as crocodiles that live in polluted environments. This hypothesis was supported by our recent work where it was observed that serum and heart lysates from crocodiles (Crocodylus palustris), have potent anti-tumour effects against prostate cancer cells, whereby 71% cell death was observed (Siddiqui et al., 2017). Following this, it was revealed that there are potentially 80 molecules detected from the crocodile. Furthermore, more than 100 potential anticancer peptides were elucidated from crocodile sera (Jeyamogan et al., 2020). In addition, the water monitor lizard was tested for its potential anticancer effects and 57 molecules were detected (Jeyamogan et al., 2019). 

Herein, we investigated the anticancer potential of two animals that have been widely utilised in the past for their medicinal properties, namely; vinegeroon scorpion (*Uropygi*) and American bullfrog (Lithobates catesbeianus). Firstly, the scorpion has been used as folk medicine in ancient civilizations of Iran and China (Liu and Ji, 2014; Mikaelian et al., 2020). Secondly, previous studies have depicted that amphibian skin possess molecules of medicinal value (Mikaelian et al., 2020). However, to our knowledge, the lysates of these remarkable species have not yet been explored. Therefore, we assessed the anticancer potential of various organ lysates of the selected animals; *Uropygi* and *L. catesbeianus* against the following cancer cells; Michigan Cancer Foundation-7 breast cancer (MCF-7), Henrietta Lacks cervical cancer (HeLa), prostate cancer (PC3) and also normal aneuploid immortal keratinocyte cell (HaCaT) using growth inhibition assay by measuring cellular growths and cytotoxicity assays by measuring lactate dehydrogenase release. 

## Material and Methods

The present study involved organ and sera collection from scorpion and frog and data obtained from subsequent experiments conducted, are hypothesis-confirming experiments and should be interpreted as such, being in the exploratory phase of research.

*Ethics committee consent and use of scorpion*

The use of scorpion and frog obtained for this study was granted by Sunway Research Ethics Committee (Research Ethics Approval Code: PGSUNREC 2019/023). Handling of the animals, species identification, anaesthesia, and dissection of the internal organs were all performed by qualified zoologist (K Sagathevan) who routinely performs these procedures. 

*Dissection*

**Figure 1:**
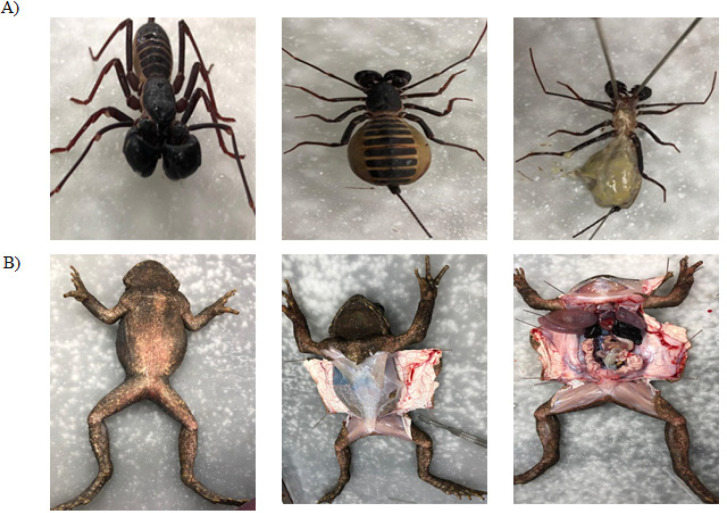
The Dissection Procedures Conducted for the Animals Used. A: *Uropygi* (Vinegaroon Scorpion) and B: *Lithobates catesbeianus* (American bullfrog).

*Uropygi* (Vinegaroon Scorpion) and *Lithobates catesbeianus* (American bullfrog) were procured from the wild and the species were identified by an expert zoologist. For the dissection of the invertebrate, *Uropygi* was deactivated by incubating on ice for 5 minutes, to immobilize. The dissections were conducted using pre-autoclaved sterile tools as described previously and various organs were collected (Figure 1) (Jeyamogan et al., 2019; Siddiqui et al., 2017). The exoskeleton, appendages and hepatopancreas were obtained from the scorpion, whereas lungs, liver, oviduct, heart, gastrointestinal tract, kidneys, gall bladder, eggs and sera, were obtained from the bullfrog. All organs and sera were stored at -80^o^C until required for further use. 

*Lysate preparation*

Subsequent to removal, the organs were homogenized using a mortar, pestle and distilled water as a solvent, 10µl/ml of protease inhibitors and Ethylene diamine tetra acetic acid (EDTA) were added to the lysate, 10 freeze-thawing cycles were performed and samples were sonicated using a Cole-Parmer cup-horn sonicator on ice. Next, the lysates were centrifuged at 14,000 x g for 80 minutes and the supernatants were collected, sterilized by filtration using a 0.22 μm filter and stored at -80^o^C for further testing. The protein concentration of all samples was elucidated using the Bradford assay (Jeyamogan et al., 2019; Siddiqui et., 2017).

*Cell culture conditions*

The prepared lysates were assessed for growth inhibitory and/or cytotoxic effects against cancer cell lines procured from the American Type of Culture Collection (ATCC). Cell lines utilized in this study include; breast adenocarcinoma cells (MCF-7) (ATCC^®^ HTB-22™), Henrietta Lack’s cervical adenocarcinoma cells (HeLa) (ATCC^®^ CCL2™) and Prostate Cancer Cells (PC3) (ATCC^®^ CRL-1435™) and a normal cell line; Human Keratinized Skin Cells (HaCaT) (ATCC^®^ PCS-200-011™). Cell lines were grown and cultured in Roswell Park Memorial Institute (RPMI-1640) supplemented with 10% foetal bovine serum (FBS), 1% L-glutamine, 1% penicillin streptomycin antibiotic and 1% Minimum Essential Media (MEM), non essential amino acid at 37^o^C, with a supply of 5% carbon dioxide and 95% humidity as previously described (Jeyamogan et al., 2019; Siddiqui et., 2017). 

*Growth inhibition assays*

Growth inhibition assays were accomplished to elucidate the growth inhibitory effect of organ lysates and sera on HeLa, MCF-7, PC3, and HaCaT cells, as previously detailed (Jeyamogan et al., 2019; Soopramanien et al., 2019). Cells were grown at 37^o^C in a 5% CO_2_ with 95% humidity, until a monolayer of up to 50% confluency was formed. Some cells were then trypsinized and cell count was determined using a haemocytometer for 50% confluency. The cells were treated with the lysates and sera from the procured animals and Bovine serum albumin (BSA) was employed as the negative control. In addition, untreated cells were also utilized as a negative control. The cells were incubated in similar conditions: 37^o^C in a 5% CO_2_ with 95% humidity, until the untreated cells became 100% confluent. The treated and untreated cells were then trypsinized with 2.5% trypsin for 15 min and subjected to trypan blue exclusion assay using a haemocytometer to determine cell viability (Jeyamogan et al., 2019; Soopramanien et al., 2019). The growth inhibition effects were established by comparing the number of viable cells of the untreated cells (control) and treated cells.

*Cytotoxicity assays*

To assess the cytotoxic effects of the organ lysate and sera against cell lines, lactate dehydrogenase (LDH) assay was conducted as described previously (Jeyamogan et al., 2019; Siddiqui et al., 2017; Soopramanien et al., 2019). For this, confluent monolayers of cancer and normal cells were grown in 96-well plates for 24 h at 37oC in a 5% CO_2_ with 95% humidity. The cells were then treated with the organ lysates and serum from the procured animals and Bovine serum albumin (BSA), was used as the negative control.cells were incubated for 24 h at 37^o^C in a 5% CO_2_ with 95% humidity. Following the incubation, the positive control was prepared by treating control cells with 0.2% Triton X-100 for 30 min at 37°C, to induce 100% cell death by rupturing the cells. The supernatant from wells containing treated and untreated cells were collected and subjected to LDH assay using Roche LDH kit reagents. The percentage of cytotoxicity was calculated as follows:

% cytotoxicity = ((Absorbancesample – Absorbancenegative control)/ (Absorbancepositive control – Absorbancenegative control)) X 100. 

The negative control consisted of cells treated with RPMI-1640 media only, while the positive control consisted of cells treated with the detergent: triton x-100.

## Results

*Organ lysates of L. catesbeianus (American bullfrog) exhibited growth inhibitory effect*

**Figure 2:**
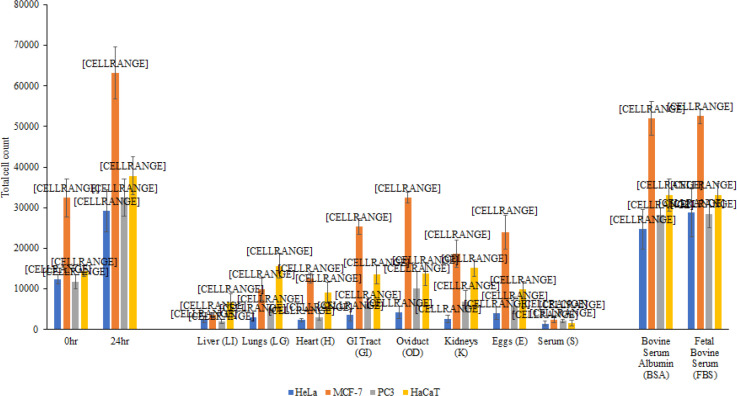
100μg/mL of Various Organ Lysates and 10% Serum of the American Bullfrog were Tested for Inhibition of Growth on HeLa, MCF-7, PC3 and HaCaT Cells. HeLa cells were grown to 50% confluency on 96-well plates (Negative Control – 0 hours) before being incubated with lysates and sera for 24 h after which viability was determined by staining with Trypan blue. The results show significant growth inhibition when compared to the control (*P < 0.05 using two sample t test and two-tailed distribution). The controls, BSA and FBS) did not affect cell proliferation. The results are representative of several experiments performed in duplicate and presented as the mean ± standard error

Lungs, liver, oviduct, gastrointestinal tract, heart, kidneys, eggs lysates, and sera exhibited significant growth inhibitory effect with P value 0.023, 0.022, 0.024, 0.024, 0.022, 0.022 and 0.020 respectively against HeLa cells, by inhibiting growth at 100%, and the cell counts following treatment were lower than the initial cell count at 0 hour (Figure 2A). This is also supported by representative microscopic images, which depict that treated cells were less confluent as compared to the negative control (Figure 2B). Similar results were observed when the lysates and serum from bullfrog were tested against MCF-7 cells. To this end, organ lysates and serum exhibited 100% growth inhibitory effects against all cell lines (Figure 2A) and the results were further supported by the microscopic images (Figure 2B). Moreover, the lysates and serum exhibited 100% growth inhibitory effects against PC3 cells, as depicted by the cell count post treatment being lower than the initial cell count at 50% confluency (Figure 2A) and these results were corroborated by microscopic images (Figure 2B). Lysates and serum from the American bullfrog also affected the growth of the normal HaCaT cells by significantly inhibiting more than 90% of cell growth, with P value 0.0011, 0.016, 0.0012, 0.0051, 0.0044, 0.0051, 0.0015 and 0.00014 respectively (Figure 2).

*Several lysates from Uropygi (Vinegaroon Scorpion) exhibited growth inhibitory effect against cancer and normal cells*

**Figure 3:**
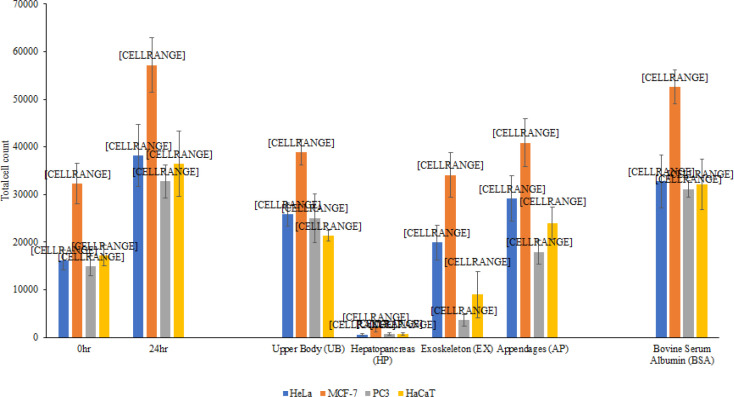
100μg/mL of Various Organ of the Vinegaroon Scorpion were Tested for Inhibition of Growth on HeLa, MCF-7, PC3 and HaCaT Cells. In brief, cells were grown to 50% confluency (Negative Control – 0 hours) before being incubated for 24 h (100% confluency) after which viability was determined by staining with Trypan blue. Hepatopancreas lysates show significant growth inhibition when compared to the control (*P < 0.05 using two sample t test and two-tailed distribution). The control, BSA (albumin, bovine serum) did not affect cell proliferation. The results are representative of several experiments performed in duplicate and presented as the mean ± standard error

The upper body, hepatopancreas, exoskeleton and appendages lysates from scorpion exhibited growth inhibitory effects against HeLa cancer cells by 56.2, 100, 82.1 and 41 % respectively, with P values 0.155, 0.056, 0.095 and 0.45 (Figure 3). The lysates from scorpion also inhibited the growth of MCF-7 cells by 73.4, 100, 92.6 and 65.5 % respectively with P values 0.061, 0.0023, 0.11 and 0.069 (Figure 3). The lysates inhibited the growth of PC3 by 43.3, 100, 100 and 83.1 % respectively, with P values 0.79, 0.025, 0.013 and 0.075 (Figure 3). However, the lysates also significantly inhibited the growth of normal HaCaT cells by 78.4, 100, 100 and 64.7% respectively, with P values 0.016, 0.031, 0.023 and 0.036 (Figure 3). The results were corroborated by representative microscopic images as the cells treated with the effective lysates were not as confluent as the untreated cells .

Several lysates from *L. catesbeianus* (American bullfrog) exhibited cytotoxic effect against cancer and normal cells.

**Figure 4:**
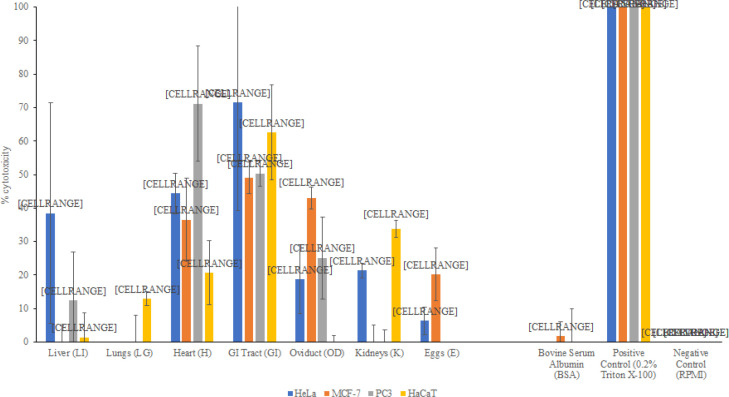
100μg/mL of Various Organ Lysates of the American Bullfrog were Tested for Cytotoxic Effects on HeLa, MCF-7, PC3 and HaCaT Cells, Using Cytotoxicity Assays by Measuring the Release of Lactate Dehydrogenase. GI Tract lysates show potent cytotoxicity. Liver, Heart, Oviduct and Kidney lysates show partial cytotoxicity. Egg and Lung lysates do not show cytotoxicity. The control, BSA (albumin, bovine serum) did not affect cell viability, while Triton-x 100 killed 100% cells. The results are representative of several experiments performed in duplicate and presented as the mean ± standard error

Liver, Heart, and gastrointestinal tract lysates from American bullfrog exhibited 38.5, 44.4, and 71.6% cytotoxicity towards HeLa cells, with P values 0.20, 0.19 and 0.12 respectively, while the remaining lysates; lungs, oviduct, kidneys and eggs exhibited limited to no cytotoxic effects against HeLa cells (Figure 4). Lysates prepared from heart, gastrointestinal tract and oviduct from American bullfrog exhibited 36.5, 49.1 and 43.0 % cytotoxic effect against MCF-7 cells with P values 0.19, 0.018 and 0.020 respectively, while the remaining lysates; liver, lungs, kidney and eggs exhibited limited to no cytotoxic effects on MCF-7 cells (Figure 4). However, only heart and gastrointestinal tract lysates from American bullfrog exhibited 71.1 and 50.4% cytotoxic effect against PC3 cells with P values 0.086 and 0.18, while the remaining; liver, lungs, oviduct, kidney, and eggs lysates did not exhibit notable cytotoxicity towards PC3 cells (Figure 4). Therefore, only heart and gastrointestinal tract lysates prepared from American bullfrog exhibited more than 30% cytotoxic effect against all the cancer cell lines; HeLa, MCF-7 and PC3 used, while liver and oviduct lysates were cytotoxic toward single cancer cell lines; HeLa and MCF-7 cells respectively. However, only gastrointestinal tract and kidneys lysates were cytotoxic against HaCaT cells by 62.6 and 33.8% with P values 0.088 and 0.012 respectively while liver, lungs, heart, oviduct, and eggs lysate exhibited limited cytotoxicity against HaCaT cells (Figure 4). Thus, gastrointestinal tract lysate was cytotoxic to both normal and cancerous cells, while heart lysate was cytotoxic to cancer cells, leaving normal cells unaffected. 

*Several lysates from Uropygi (Vinegaroon Scorpion) exhibited cytotoxic effect against cancer and normal cells*

**Figure 5:**
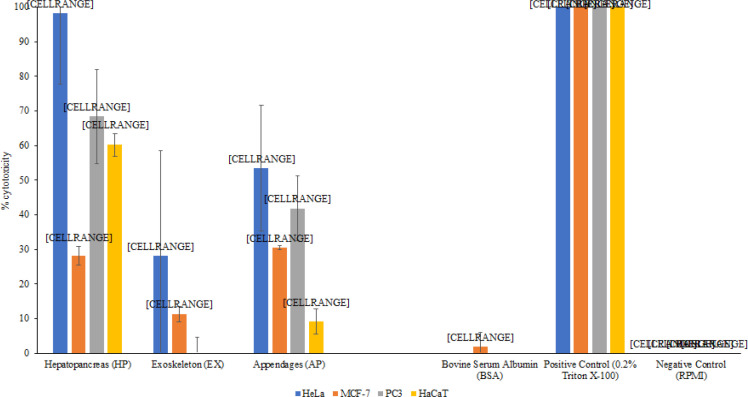
100μg/mL of Various Organ Lysates of the Vinegaroon Scorpion were Tested for Cytotoxic Effects on HeLa, MCF-7, PC3 and HaCaT Cells, Using Cytotoxicity Assays by Measuring the Release of Lactate Dehydrogenase. Cytotoxicity was exhibited by Exoskeleton, Appendages and Hepatopancreas lysates of which the later showed significant cytotoxicity. The control, BSA (albumin, bovine serum) did not affect cell viability, while Triton-X 100 killed 100% cells. The results are representative of several experiments performed in duplicate and presented as the mean ± standard error

Hepatopancreas and appendages lysate from scorpion exhibited 98.3 and 53.5% cytotoxic effect against HeLa cells (P value 0.043 and 0.081), while exoskeleton lysate exhibited limited cytotoxicity of 28.1% against HeLa cells (P value 0.24) (Figure 5). While the lysates from scorpion exhibited cytotoxicity towards HeLa cells, Hepatopancreas, exoskeleton and appendages lysates exhibited 28.2, 11.4 and 30.5 % cytotoxicity with P value 0.048, 0.23 and 0.089 respectively against MCF-7 cells which indicate for limited cytotoxic effect (Figure 5). Thus, none of the lysates were cytotoxic to MCF-7 cells. However, hepatopancreas and appendages lysates from scorpion exhibited 68.4 and 41.8% cytotoxicity against PC3 cells with P value 0.049 and 0.082 respectively, while exoskeleton lysate did not exhibit any cytotoxic effect against PC3 cells (Figure 5). Only hepatopancreas lysate from scorpion exhibited 60.2% significant cytotoxicity towards against HaCaT cells (P value 0.0045), while both exoskeleton and appendages lysates from scorpion only exhibited no cytotoxic effect against the normal HaCaT cells (Figure 5). Therefore, the cytotoxicity results indicated that hepatopancreas lysates were cytotoxic to both normal and cancer cells except for MCF-7 cells, while appendages lysates exhibited cytotoxicity towards HeLa and PC3 cancer cells. 

## Discussion

To date, there have been no studies conducted to elucidate the anticancer activity of frog and scorpion lysates and sera. Nonetheless, several studies have been accomplished in our laboratory addressing our hypothesis that: “*animals living in polluted environments may have anticancer activities*” (Jeyamogan et al., 2019; Jeyamogan et al., 2020, Siddiqui et al., 2017; Soopramanien et al., 2019). These studies have shown that animals such as crocodiles reveal potent anti-cancer effects. In this study, we determined potential anticancer activity of organs from two animals: a vertebrate American bullfrog: *L. catesbeianus* and an invertebrate scorpion: *Uropygi*. Those animals were selected to test for potential anticancer activity of their organ lysates as they are known to thrive in environments infested with pathogenic microorganisms and pollutants which are considered as common cancer risk factors in humans and they are also known to feed on insects and bacteria-infested decaying matter. But still, those animals are able to thrive in those environments (Jeyamogan et al., 2017), suggesting the presence of molecules/mechanisms to counter carcinogenic materials. 

Our results revealed that lysates; liver, lungs, heart, gastrointestinal tract, oviduct, kidneys and eggs and serum from the bullfrog *L. catesbeianus*, exhibited growth inhibitory effects against all cell lines tested. However, the heart lysate exhibited cytotoxic effects against cancer cells, leaving the normal HaCaT cells unaffected. Besides the gastrointestinal and heart lysates, liver and oviduct lysates exhibited cytotoxic effects towards HeLa and MCF-7 cells respectively. Our findings support earlier studies on the skin of frogs which showed that extracts from frog skin possess medicinal activities. In 2012, van Zoggel et al., found two antimicrobial α-helical cationic peptides (dermaseptins B2 and B3) in the skin secretions of the South American tree frog, Phyllomedusa bicolor which inhibited the proliferation of human cancer cells by inhibiting cell colony formation in soft agar (van Zoggel et al., 2012). Furthermore, Bufalin (BF), a molecule isolated from the venom of Chinese toad: Bufo gargarizans (Asiatic toad) is known to exhibit properties against cancer cell lines without significantly affecting normal cells (lan et al., 2019; Takai et al., 2012; Yin et al., 2012). Thus, it was speculated that lysates from the American bullfrog in this study may potentially exhibit similar mechanism of action, however, this needs to be established in future studies.

In addition, our results revealed that the hepatopancreas and exoskeleton lysates of the scorpion exhibited more that 60% growth inhibitory effect towards cancer and normal cells. While the upper body lysate exhibited more than 60% growth inhibitory effect against HeLa, MCF-7 and HaCaT cells, without having notable growth inhibitory effects against PC3 cells, the lysates from appendages inhibited the growth of MCF-7, PC3 and HaCaT cells without notably affecting the growth of HeLa cells. However, the cytotoxicity results indicated that hepatopancreas lysates were cytotoxic to both normal and cancer cells except for MCF-7 cells, while appendages lysates only exhibited cytotoxicity towards HeLa and PC3 cancer cells. Of note, the exoskeleton and appendages lysate from the scorpion were not toxic to human cells. Lysates that were toxic to human cells could be employed using targeted cancer therapy, whereas the lysates that were not toxic to human cells, need to be investigated further, and this is very encouraging, as most cancer drugs tend to have severe side effects and toxicity.

To our knowledge, for the first time, the antitumor potential of organ lysates and sera from both the bullfrog and the scorpion were assessed. It was observed that the serum and gastrointestinal tract of the bullfrog exhibited effects against cancer cell lines, but it is unclear whether the anticancer potential resulted from the lysate and sera or from the microbiota of the animals tested in this study. Since the gastrointestinal tract was processed as a whole in this study, it is possible that the gut microbiota might be responsible for the anticancer potential of the former. Therefore, further studies should be conducted to assess whether the anticancer potential is mainly due to the gastrointestinal lysate or the secretions from the gut microbiota. Since the gut microbiota is known to be beneficial to its host (Heyde and Ruder, 2015; Louis et al., 2014; Kang et al., 2017; Kelly et al., 2017; Zhou et al., 2017). Moreover, studies have shown that the metabolites synthesized by the gut microbiota are transported to the brain through the blood (Belkaid and Hand, 2014; Clos-Garcia et al., 2019). Thus, hinting that those active molecules in the serum of the frog might be synthesized by the gut microbiota. Hence, the gut microbiota of these animals should be assessed for their anticancer potential. 

In order to identify the molecules with potential anticancer activity present in the crude lysates of the scorpion and bullfrog, liquid chromatography-mass spectrometry (LC-MS) will be conducted to identify the molecules present in the lysates. Moreover, the identified molecules need to be tested individually and in combination, for potential anti-cancer activity as described in this study. Since the crude lysates expressed anticancer properties, isolation, and identification of the molecules present in those crude lysates might lead to the discovery of novel molecules with anticancer potentials as an alternative chemotherapeutic agent, to treat the rising number of cancer patients. 

In summary, these findings suggest that the scorpion and the frog may produce anticancer molecules that are effective against various cancer cells and it is anticipated that various potentially innovative anticancer molecules will be identified in future studies. The lysates tested showed potent anticancer effects albeit some lysates displayed cytotoxic effects against normal human cells. The molecular identity of active molecules coupled with their mechanism of action will need to be elucidated in future studies. Nonetheless, intensive research in the coming years is required to determine the translational value of these findings 

## Declarations

Ethics approval: The use of scorpion and frog obtained for this study was granted by Sunway Research Ethics Committee (Research Ethics Approval Code: PGSUNREC 2019/023).
